# Formulating a national position statement and guide on modern theranostics in the Philippines

**DOI:** 10.22038/AOJNMB.2023.72838.1508

**Published:** 2024

**Authors:** Patricia A. Bautista-Peñalosa, Francis Gerard M. Estrada, Emerita A. Barrenechea, Teofilo O.L. San Luis, Jr

**Affiliations:** 1 St. Luke’s Medical Center - Global City, Philippines; 2 Our Lady of Mt. Carmel Medical Center, Pampanga, Philippines; 3 Veterans Memorial Medical Center, Quezon City, Philippines; 4 St. Luke's College of Medicine, Quezon City, Philippines

**Keywords:** PSNM, Developing country Theranostics accreditation Patient safety

## Abstract

Barriers to the establishment of advanced technologies in developing countries were overcome when modern theranostics pertaining to the use of Ga-68 and Lu-177 PSMA and DOTATATE were first offered to patients in the Philippines in early 2018. However, significant growth was not experienced at St. Luke’s Medical Center for five years and lutetium was not yet distributed to other institutions by a radiopharmaceutical supplier. Due to the relative novelty and rapid expansion of theranostics worldwide, position statements were released by the Australasian Association of Nuclear Medicine Specialists, European Association of Nuclear Medicine, Society of Nuclear Medicine and Molecular Imaging, and International Atomic Energy Agency primarily to uphold patient safety and ensure a level of standard among its practitioners. Subsequently in the latter half of 2022, these were adopted and modified according to what is feasible and applicable locally within the Philippine Society of Nuclear Medicine, considering the current status and future possibilities. Different representatives were involved, and several groups were mobilized for successful implementation. A liability clause was incorporated to discourage unprofessional acts.

## Introduction

Introducing modern theranostics pertaining to the use of Ga-68 and Lu-177 PSMA for metastatic prostate cancer and Ga-68 and Lu-177 DOTATATE for neuroendocrine tumors in a developing country like the Philippines is not an easy feat. Advanced technologies are not prioritized in government hospitals. Expensive procedures and treatments are not subsidized or covered by the Philippine Health Insurance Corporation (PhilHealth) and most health maintenance organizations (HMOs). Novel equipment is not voluntarily bought and simply handed to physicians in private hospitals. 

 Considering these factors, excellent training is crucial to be able to convince decision-makers to invest in theranostics. Hence, after being mentored by Prof. Dr. Richard Baum in a high-volume theranostics center in late 2016, Dr. Patricia Bautista was able to spearhead the establishment of theranostics at St. Luke’s Medical Center in the Philippines in early 2018 (1). 

 This paper aims to give an overview of the Philippine scenario, to highlight the leadership role of the respected international organi-zations in upholding high-quality patient care, and to illustrate the process of adopting and modifying these international guidelines into a national position statement. 

***Philippine Scenario***

 From the first year of 2018 with a total peptide receptor radionuclide therapy (PRRT) and prostate-specific membrane antigen (PSMA) radioligand therapy (PRLT) census of 9 therapies at St. Luke’s Medical Center – Quezon City and 10 therapies at St. Luke’s Medical Center – Global City, there was an increase to 32 and 64, respectively, in 2019. However, there was no increasing trend in the years that followed. In Quezon City, there were 1.5 therapies a month on average for five years whereas in Global City, there were 3.4. Clearly, the demand was not high.

 At this time, no other institution was capable of offering theranostics. However, with the readiness of KHealth Corporation, a Korean-owned radiopharmaceutical distributor in the Philippines, to sell Lu-177 PSMA initially to interested centers, straightforward accessibility was imminent. 

***International Guidelines***

 In February 2021, the Australasian Association of Nuclear Medicine Specialists (AANMS) was the first to come up with a position statement and declare that “the development of skills and expertise in theranostics is necessary for its safe and effective use in a clinical setting.” According to the group, “practical experience is critical to optimal service delivery and requires exposure to a sufficient volume,” i.e., >50 therapy administrations for general accreditation and >120 therapy administrations for advanced accreditation (2-3). 

 More than a year later, the Joint EANM, SNMMI and IAEA Enabling Guide: How to set up a theranostics centre was published in April 2022. The high importance of additional training of existing board-certified nuclear medicine specialists, as well as the incorporation of theranostics into the current training curricula, was stressed. Experienced or high-volume training centers were identified. 

 The shift towards a more participatory or principal rather than passive role in patient management was recognized. The involvement of national and international societies was encouraged. Careful preparation and planning as key to successful implementation was emphasized (4). 

***Formulation of the PSNM Position Statement***

 Following the release of the two publications from four esteemed and credible organizations namely the AANMS, European Association of Nuclear Medicine, Society of Nuclear Medicine and Molecular Imaging, and International Atomic Energy Agency, the Philippine Society of Nuclear Medicine (PSNM) Position Statement and Guide on the Practice of Modern Theranostics in the Philippines was developed in July 2022. The current practitioners of theranostics, the incumbent PSNM President, another PSNM officer, the Philippine Nuclear Research Institute (PNRI) liaison, and two respected original Fellows of the PSNM were invited for a meeting. A primer on the status of theranostics in the country, in the region, and worldwide was written to provide a landscape and set the bar. A dialogue with the PNRI, which is the national regulatory body for the use of radioactive material (RAM), was held to enlighten them on the practice of theranostics in the country and to reiterate the requirement of “at least 2 years of relevant clinical training and experience” for the approval of a RAM license, in accordance with the Code of PNRI Regulations Part 13 (5). After several discussions, the PSNM Position Statement was finalized and approved by the PSNM Officers and Board of Directors and announced to the members and the public in August 2022 (6).

***Highlights of the PSNM Position Statemen*** t

 For better understanding of the need for the PSNM Position Statement, it was explicitly stated that modern theranostics is currently not included in the nuclear medicine residency and fellowship training curricula in the Philippines. Furthermore, differences with radioactive iodine (RAI) were enumerated to correct the misconception that practicing Ga-68 and Lu-177 theranostics, more so establishing it, is as simple as ordering a radiopharmaceutical and giving it to the patient, and therefore, no further training is necessary. Even if the radio-pharmaceutical is ordered from a local distributor and not synthesized using a hospital’s own equipment, logistics is still more complex as the timing of the arrival of the radiopharmaceutical, administration of the pre-medications, and post-therapy scan around 24 hours later must be closely coordinated. The following table 1 shows the distinction in many aspects, excluding radiation precautions, which are less stringent in PRRT and PRLT compared to RAI therapy (7-10). 

**Table 1:** Differences between RAI and Modern Theranostics

	**Radioactive iodine**	**Ga-68 and Lu-177**
Discovery	1940s	2000, 2013
Training curriculum	yes	no
Patient characteristics	young to middle-aged with few co-morbidities	elderly, end-stage
Screening	minimal	battery of laboratory and imaging tests
Administration	usually once, oral	multiple times, IV
Follow-up	infrequent	frequent
Multidisciplinary team	endocrinologist, surgeon	(e.g. for PRLT) urologist, radiation oncologist, medical oncologist, hematologist, nephrologist, pain specialist
Logistics	local supplier	international supplier, PNRI, Accounting, Bureau of Customs, Admitting, production team, nursing unit
Cost of the radio-pharmaceutical	PHP 35,000-45,000 (cost for 200 mCi I-131)	PHP 370,000-500,000 (cost for 200 mCi Lu-177 DOTATATE or PSMA)

 For the Objectives, apart from promoting patient safety and standardization of competencies, the PSNM Position Statement was created to underscore the responsibility of the theranostician in the multidisciplinary team with an active role in patient management. The theranostician is expected to be more visible in multidisciplinary conferences not only to present images; he or she must also be capable of readily identifying good candidates for PRRT or PRLT, or firmly refusing to accept referrals if the treatment is not deemed beneficial. 

 Moreover, the theranostician must be able to interpret laboratory test results, understand treatment-related risks, and diagnose and address side effects, as well as satisfactorily answer the queries of the patient, relatives, and other members of the multidisciplinary team (11). Due diligence and care must be exercised; otherwise, imprudent patient selection and unsystematic actions resulting in poor outcome can discredit the specialty of nuclear medicine, particularly in light of other specialists and patients who are still not receptive to theranostics. 

 Because healthcare expenditure is shouldered mostly by patients in the Philippines and the lutetium-177 is paid for in advance to forestall any last-minute cancellations, minimization of wastage of radiopharmaceuticals and finances was distinguished as an objective as well. If patients are not screened meticulously, USD 6,800-9,200 can potentially be wasted.

 Two levels of accreditation were decided on, similar to the AANMS Position Statement. However, the minimum requirement for general accreditation was reduced to 25 therapy administrations, which is equivalent to the requirement for RAI therapy in the existing PSNM training curriculum, in order to be as inclusive as possible without compromising the welfare of patients and the integrity of the PSNM. The high census for the advanced accreditation was maintained because the presence of an advanced theranostician onsite is one of the criteria for a theranostics center to become an accredited training center. A minimum census of 35 PRRT/PRLT adminis-trations a year was also specified for eligibility as an accredited theranostics training center to ensure that centers with little or hardly any experience at all would not claim undue expertise and become a training ground. The Theranostics Committee and the Accreditation Committee of the PSNM were assigned to evaluate applicants.

 To deter those carrying out false advertisement, misrepresentation or any form of unethical practice, a liability clause was incorporated in the PSNM Position Statement. 

 The Ethics Committee of the PSNM, which is composed of the current and former presidents, was tasked to look after possible unprofessional practices. 

***Current Status and Implementation***

 As of August 1, 2023, there are 3 advanced and 9 general theranosticians in the Philippines who are affiliated in various hospitals. In terms of the present demand, these are sufficient to cater to the patients who can avail themselves of theranostics services. St. Luke’s Medical Center is still the only accredited theranostics training center locally and the only hospital with synthesizing capabilities through its supplier, ITM Isotope Technologies Munich SE. The other hospitals or centers are currently dependent on Khealth Corporation to sell Lu-177 PSMA. Two other local suppliers in addition to Novartis may soon provide commercial options.

 The PSNM Position Paper is enforced successfully by the Theranostics Committee, Accreditation Committee, and the PSNM Board. Every PSNM member is asked to comply with its provisions. Self-promotion without substanti-ation of claims and acts unbecoming of a true professional are discouraged. Shortcuts such as merely reviewing cases on a computer or watching You Tube videos are not tolerated. 

 As the field of theranostics is rapidly evolving, it is acknowledged that the PSNM Position Statement may be up for revision every year or as deemed essential. Alongside this, the training curriculum for Nuclear Medicine residents and fellows in the Philippines is currently being revised to include a rotation in Urology and to lengthen the rotation in Internal Medicine, as well as add more engagement in multi-disciplinary meetings. Hopefully, further radiopharmaceutical wastage, financial loss, and untimely death of a patient due to improper handling will be prevented. 
